# Biochemical and molecular characterization of a novel glycerol dehydratase from *Klebsiella pneumoniae* 2e with high tolerance against crude glycerol impurities

**DOI:** 10.1186/s13068-023-02427-8

**Published:** 2023-11-16

**Authors:** Zifeng Lin, Yuting Xiao, Lu Zhang, Le Li, Congying Dong, Jiangshan Ma, Gao-Qiang Liu

**Affiliations:** 1 Hunan Provincial Key Laboratory of Forestry Biotechnology, Central South University of Forestry and Technology, Changsha, 410004 China; 2 International Cooperation Base of Science and Technology Innovation on Forest Resource Biotechnology, Central South University of Forestry and Technology, Changsha, 410004 China; 3 Microbial Variety Creation Center, Yuelushan National Laboratory of Seed Industry, Changsha, 410004 China

**Keywords:** Glycerol dehydratase, *Klebsiella pneumoniae* 2e, Enzymatic activity, High tolerance, Crude glycerol impurities, Coil structure, Protein flexibility

## Abstract

**Background:**

The direct bioconversion of crude glycerol, a byproduct of biodiesel production, into 1,3-propanediol by microbial fermentation constitutes a remarkably promising value-added applications. However, the low activity of glycerol dehydratase, which is the key and rate-limiting enzyme in the 1,3-propanediol synthetic pathway, caused by crude glycerol impurities is one of the main factors affecting the 1,3-propanediol yield. Hence, the exploration of glycerol dehydratase resources suitable for crude glycerol bioconversion is required for the development of 1,3-propanediol-producing engineered strains.

**Results:**

In this study, the novel glycerol dehydratase 2eGDHt, which has a tolerance against crude glycerol impurities from *Klebsiella pneumoniae* 2e, was characterized. The 2eGDHt exhibited the highest activity toward glycerol, with *K*_*m*_ and *V*_*m*_ values of 3.42 mM and 58.15 nkat mg^−1^, respectively. The optimum pH and temperature for 2eGDHt were 7.0 and 37 °C, respectively. 2eGDHt displayed broader pH stability than other reported glycerol dehydratases. Its enzymatic activity was increased by Fe^2+^ and Tween-20, with 294% and 290% relative activities, respectively. The presence of various concentrations of the crude glycerol impurities, including NaCl, methanol, oleic acid, and linoleic acid, showed limited impact on the 2eGDHt activity. In addition, the enzyme activity was almost unaffected by the presence of an impurity mixture that mimicked the crude glycerol environment. Structural analyses revealed that 2eGDHt possesses more coil structures than reported glycerol dehydratases. Moreover, molecular dynamics simulations and site-directed mutagenesis analyses implied that the existence of unique Val744 from one of the increased coil regions played a key role in the tolerance characteristic by increasing the protein flexibility.

**Conclusions:**

This study provides insight into the mechanism for enzymatic action and the tolerance against crude glycerol impurities, of a novel glycerol dehydratase 2eGDHt, which is a promising glycerol dehydratase candidate for biotechnological conversion of crude glycerol into 1,3-PDO.

**Supplementary Information:**

The online version contains supplementary material available at 10.1186/s13068-023-02427-8.

## Background

Crude glycerol is the main byproduct from biodiesel production, and it is generated in enormous quantities in the biodiesel industry [1, 2]. The presence of a range of impurities, such as methanol, inorganic salts, and residual free fatty acids, in crude glycerol prevents further industrial utilization without an expensive purification process, which causes it to be treated as waste in biodiesel plants instead of valuable resource [3, 4]. Direct utilization of crude glycerol as the substrate for the production of important chemicals, such as 1,3-propanediol, 2,3-butanediol, 1,2-propanediol, and succinic acid, offers a remarkably promising approach for value-added applications [5, 6]. Among those, the bioconversion of crude glycerol into 1,3-propanediol (1,3-PDO) by microorganisms has gained extensive attention, because it is an important bulk chemical with many industrial uses, especially in the synthesis of the biodegradable plastic polytrimethylene terephthalate (PTT) [7, 8]. *Klebsiella* genus strains, which show high tolerance to the crude glycerol environment, are promising candidates for 1,3-PDO production from crude glycerol [9, 10]. However, the yield of 1,3-PDO from crude glycerol by this genus was still lower than that of pure glycerol, which was primarily caused by the presence of impurities [3]. The low activities of key enzymes involved in glycerol metabolism, which are caused by the impurities in crude glycerol, are one of the main factors affecting the yield of 1,3-PDO from crude glycerol [11, 12].

In the glycerol metabolism pathway of *Klebsiella* species, glycerol dehydrogenase, glycerol dehydratase, and 1,3-PDO oxidoreductase are the major enzymes associated with 1,3-PDO production [13]. Among these enzymes, glycerol dehydratase (GDHt, EC 4.2.1.30), which initiates the first reaction in the reductive branch of the 1,3-PDO synthetic pathway, is one of the key and rate-limiting enzymes for 1,3-PDO production [13]. In the *Klebsiella* genus strain, glycerol dehydratase catalyzes the coenzyme B_12_-dependent dehydration reaction of glycerol generating 3-hydroxypropionaldehyde (3-HPA), which is further reduced to the fermentation product 1,3-PDO by 1,3-propanediol oxidoreductase [14]. A number of studies have demonstrated that the activity of glycerol dehydratase during the catalytic process is determined by the activity of the corresponding reactivating factor, the rate of adenosylcobalamin regeneration, and the fermentation environment [15–17]. During crude glycerol fermentation, glycerol dehydratase activity is susceptible to be impacted by the impurities, resulting in low yields of 1,3-PDO [18]. Numbers of *Klebsiella* genus strains have been used for crude glycerol fermentation, and the main focus is typically on the tolerance of the strain toward impurities and optimization of the fermentation conditions, while there was less concern about glycerol dehydratase activity during fermentation [19–21]. The crystal structure and the mechanisms for catalysis, suicide inactivation, and reactivation of B_12_-dependent glycerol dehydratase have been extensively explored [16, 17, 22]. In addition, the improvement of the glycerol dehydratase stability and activity by site-mutagenesis has been reported [23]. Nevertheless, studies on natural glycerol dehydratase resources with excellent performance, especially with high resistance to crude glycerol impurities, have rarely been reported. The discovery of glycerol dehydratase resources with high tolerance toward the crude glycerol environment and its molecular mechanism would greatly contribute to the development of engineered strains for 1,3-PDO production from crude glycerol.

In our previous study, we characterized the newly isolated strain *K*. *pneumoniae* 2e, which has the capacity to produce 1,3-PDO efficiently from crude glycerol [24]. The enzymatic activity of glycerol dehydratase (2eGDHt) from this strain was almost unaffected during crude glycerol fermentation, and it showed high tolerance toward the crude glycerol environment [24]. However, the detailed molecular and biochemical characteristics of this enzyme remain unknown. In this study, a sequence analysis of 2eGDHt was first conducted. Then, recombinant 2eGDHt was obtained, and its biochemical characteristics, including the optimum pH and temperature and substrate specificity, were investigated. Subsequently, the tolerance of 2eGDHt toward crude glycerol impurities was studied. Furthermore, the molecular mechanism for the tolerance of 2eGDHt toward crude glycerol impurities was explored with homology modeling, molecular dynamics (MD) simulations, and site-directed mutagenesis. To the best of our knowledge, this is the first report on the biochemical and molecular characterization of a glycerol dehydratase from *Klebsiella* species with high tolerance against crude glycerol impurities.

## Results and discussion

### Sequence analysis of 2eGDHt

**Fig. 1:**
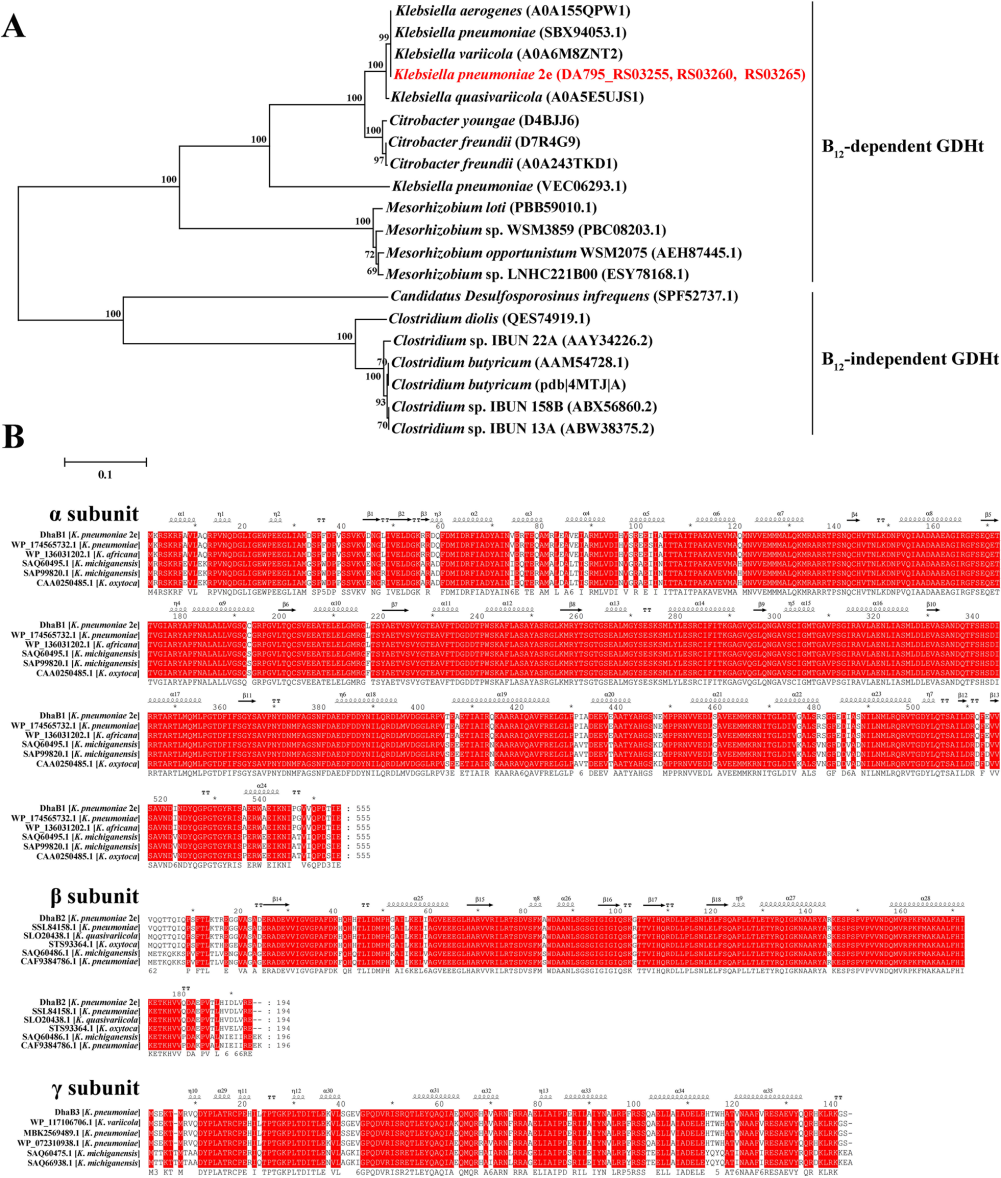
Bioinformatic analysis of the 2eGDHt sequence. **A** Phylogenetic tree comparison of the amino acid sequence of 2eGDHt with those of reported glycerol dehydratases. The tree was constructed with the neighbor-joining algorithm (1000 bootstrap trials) using MEGA 5.1. **B** Multiple sequence alignment of the amino acid sequences of 2eGDHt and reported glycerol dehydratases from *Klebsiella* genus strains using Clustalx and GeneDoc software

In our previous study, the whole genomic analysis of *K*. *pneumonia* 2e revealed that its glycerol dehydratase (2eGDHt) enzyme consisted of three subunits (*α*, *β*, and *γ*) encoded as *dhaB*1 (AYO66109.1), *dhaB*2 (AYO66110.1), and *dhaB*3 (AYO66111.1), respectively, with a total length of 2693 bp [24]. To investigate the evolutionary position of 2eGDHt, 19 glycerol dehydratases from the B_12_-dependent and B_12_-independent families were selected for protein sequence-based phylogenetic tree construction. As shown in Fig. 1A, the sequence of 2eGDHt fell within the glycerol dehydratase from *Klebsiella* genus strains, which belong to the B_12_-dependent glycerol dehydratase family. The common feature in the enzymatic reaction of B_12_-dependent glycerol dehydratase is that it proceeds by a radical mechanism that requires coenzyme B_12_ as a cofactor for generating highly active primary carbon radicals for catalysis [16].

To study the sequence similarity of 2eGDHt with other glycerol dehydratases from *Klebsiella* genus strains, a multiple sequence homologous alignment analysis was conducted. As shown in Fig. 1B, the protein sequences were highly conserved among glycerol dehydratases from *Klebsiella* genus strains. The sequence similarities of the α, β, and γ subunits reached 90.2%, 92.7%, and 88.6%, respectively, among those glycerol dehydratases. The catalytic amino acid residues, including Glu171, Gln142, Glu222, Gln297, and Ser363, were absolutely conserved in all seven glycerol dehydratase protein sequences compared, which was consistent with a previous study [25]. The glycerol dehydratase sequences from *K*. *pneumoniae* strains were very similar, showing only a few different amino acids. Notably, an isoleucine was located at position 744 in 2eGDHt instead of the valine found in the other two compared glycerol dehydratase protein sequences from *K*. *pneumonia*.

### Heterologous expression and purification of the recombinant 2eGDHt

**Fig. 2:**
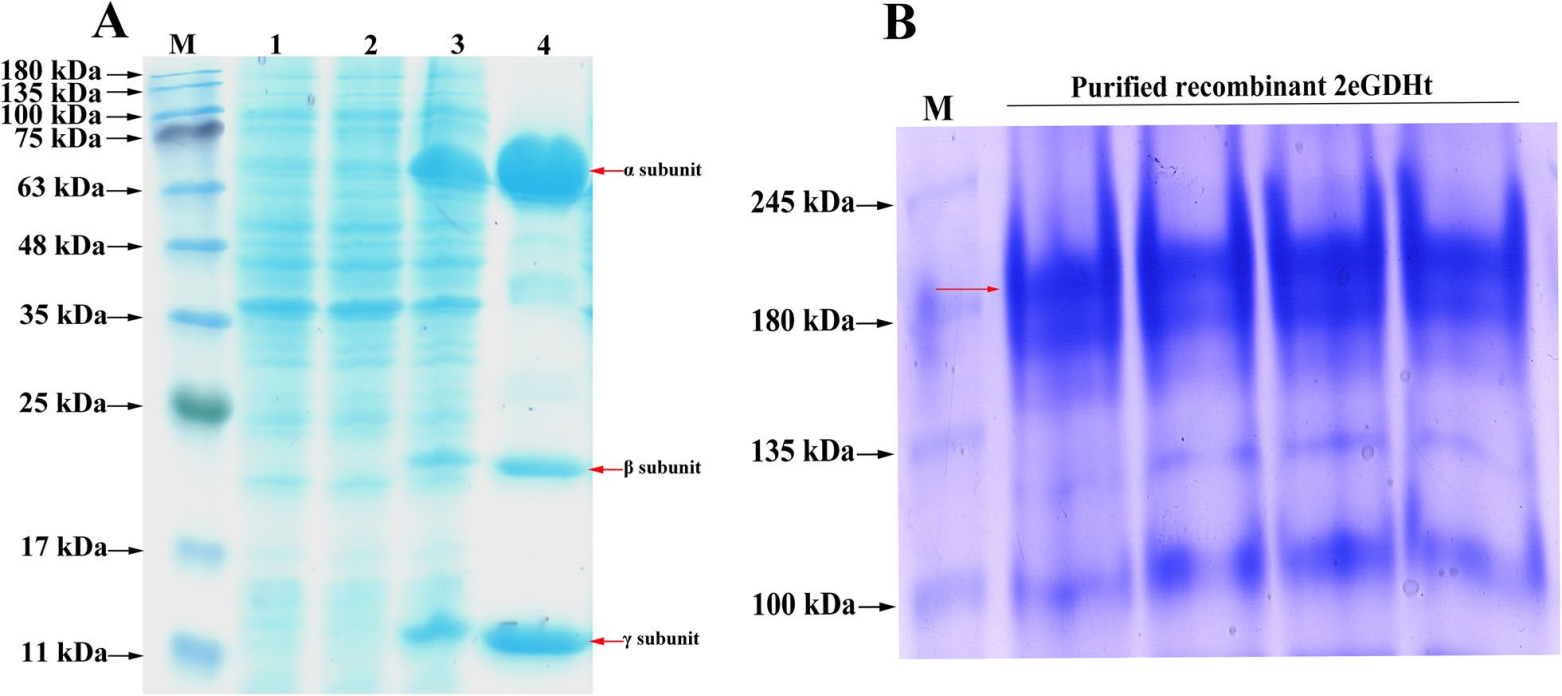
Analysis of the recombinant 2eGDHt overexpressed from *E*. *coli* BL31 (DE3) by SDS-PAGE and Native-PAGE. **A** SDS-PAGE analysis. Lane M, molecular weight marker; Lane 1, total cellular proteins expressed from *E*. *coli* BL31 (DE3) harboring empty vector induced by IPTG; Lane 2, total cellular proteins expressed from *E*. *coli* BL31 (DE3) without inducer; Lane 3, total cellular proteins expressed from *E*. *coli* BL31 (DE3) induced by IPTG; Lane 4, purified recombinant 2eGDHt. **B** Native-PAGE analysis of purified recombinant 2eGDHt

The full-length *dhaB* gene-encoding 2eGDHt was amplified from the genomic DNA of *K*. *pneumoniae* 2e by PCR and cloned and inserted into the pET-28b vector. The recombinant 2eGDHt was overexpressed as a soluble protein after induction with 0.8 mM IPTG and purified to homogeneity by affinity chromatography on a Ni–NTA resin column. As shown in Fig. 2A, three bands with approximate molecular weights of 60 kDa, 21 kDa, and 16 kDa, were observed with SDS-PAGE of the purified recombinant 2eGDHt, which was consistent with molecular weights deduced from the amino acid sequences of the three subunits. It has been reported that B_12_-dependent glycerol dehydratase from bacterial strains exists as a dimer of heterotrimers (*αβγ*)_2_ in the natural state [26, 27]. Native-PAGE analysis of the purified recombinant 2eGDHt revealed a single band with an approximate molecular weight of 190 kDa (Fig. 2B), suggesting that it naturally presents as a dimer of heterotrimers. These results indicated that recombinant 2eGDHt was successfully expressed and purified.

### Substrate specificity of 2eGDHt

To explore the substrate specificity of 2eGDHt, a number of different alcohol substrates, including glycerol, 1,2-propanediol (1,2-PDO), methanol, butanol, isopropanol, and ethanol, were selected for enzymatic assays. As listed in Table 1, the enzyme exhibited activities toward those substrates with different values, indicating that it possessed broad substrate specificity. Among those substrates, 2eGDHt displayed the highest activity against glycerol with a value of 0.98 ± 0.05 U/mg, followed by 1,2-PDO, with a value of 0.79 ± 0.03 U/mg. However, it showed limited activities toward methanol, ethanol, butanol, and isopropanol. As with other reported studies, glycerol was the most suitable substrate for 2eGDHt [16]. 1,2-PDO is the physiological substrate of diol dehydratase, which is the functional enzyme of glycerol dehydratase [28, 29]. Hence, the activity of 2eGDHt against 1,2-PDO was the second highest among those substrates. The kinetic parameters of 2eGDHt for glycerol and 1,2-PDO are presented in Table 2, and the kinetic *K*_*m*_ and *V*_*max*_ values for glycerol were 3.42 mM and 58.15 nkat mg^−1^, respectively. The catalytic efficiency (*K*_*m*_/*K*_*cat*_) of 2eGDHt for glycerol reached 53.17 s^−1^ mM^−1^, which was higher than that for 1,2-PDO (46.48 s^−1^ mM^−1^).

**Table 1:** The substrate specificity of 2eGDHt

Substrate	Specific activity (U/mg)
Glycerol	0.98 ± 0.05
1,2-Propanediol	0.79 ± 0.03
1,2-Butanediol	0.64 ± 0.05
Butanol	0.53 ± 0.01
Methanol	0.58 ± 0.08
Ethanol	0.52 ± 0.02
Isopropanol	0.51 ± 0.03

The substrate specific activities were determined by incubated with each of the 0.2 M substrates and incubated for 10 min at 37 °C and pH 7.0. Results are presented as means ± standard deviation (*n* = 3)

**Table 2:** Kinetic parameters of 2eGDHt with glycerol and 1,2-PDO

Substrate	*K*_m_ (mM)	*V*_max_ (nkat mg^−1^)	*K*_cat_ (s^−1^)	*K*_cat_/*K*_m_ (s^−1^ mM^−1^)
Glycerol	3.42	58.15	181.72	53.17
1,2-PDO	3.70	106.65	172.29	46.48

Enzyme assay was performed by incubated with each of the substrates ranging from 2 to 200 mM at 37 °C and pH 7.0 for 10 min

### Effects of temperature and pH on the enzymatic activity and stability of 2eGDHt

**Fig. 3:**
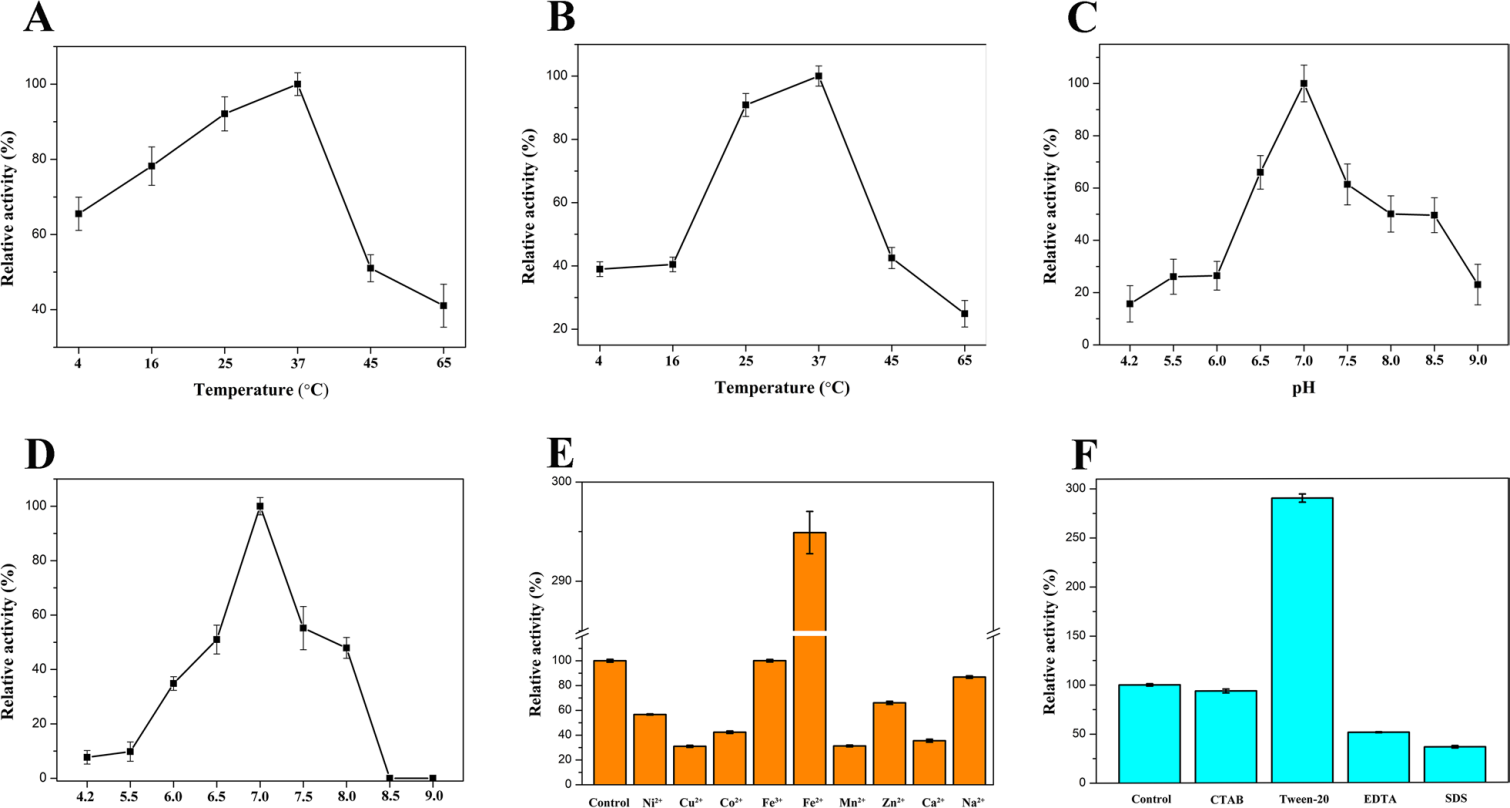
Biochemical characterization of 2eGDHt. **A** Effect of temperature on the 2eGDHt activity. The enzyme activity was determined at temperatures ranging from 4 to 65 °C at pH 7.0. **B** Effect of temperature on the 2eGDHt stability. The enzymatic activity was measured at 37 °C and pH 7.0 after preincubation at temperatures ranging from 4 to 65 °C. **C** Effect of pH on the 2eGDHt activity. The enzymatic activity was determined at pH values ranging from 4.2 to 9.0 at 37 °C. **D** Effect of temperature on the 2eGDHt stability. The enzymatic activity was measured at 37 °C and pH 7.0 after preincubation at pH values ranging from 4.2 to 9.0. **E** Effects of various metal ions on the enzymatic activity of 2eGDHt. **F** Effects of various chemical reagents on the enzymatic activity of 2eGDHt

To investigate the effect of temperature on the activity of 2eGDHt, the enzymatic activity was determined at different temperatures (4–65 °C). As shown in Fig. 3A, 2eGDHt displayed more than 40% relative activity over the range 4–65 °C, and it exhibited the maximum activity at 37 °C. The activity of 2eGDHt decreased as the temperature was increased over 37 °C with 41.0% relative activity at 65 °C. This indicated that the optimum temperature for 2eGDHt was 37 °C. The stability of 2eGDHt at various temperatures was explored after it was preincubated at temperatures ranging from 4 to 65 °C at pH 7.0 for 24 h. As shown in Fig. 3B, the enzyme was highly stable between 25 and 37 °C, and maintained more than 80% of its activity in this range. Meanwhile, it retained more than 40% activity at 16 and 45 °C, indicating strong stability in this temperature range. The optimum temperature for 2e-GDHt was similar to that reported for GDHt from *K*. *pneumoniae* strains [30]. The temperature stability for 2eGDHt ranged from 16 to 45 °C, which was a broader range than that of the reported glycerol dehydratase from the *K*. *pneumoniae* strain [30].

The effect of pH on enzyme activity was assayed at various pH values ranging from 4.2 to 9.0. As shown in Fig. 3C, 2eGDHt retained more than 40% relative activity at pH values ranging from 6.5 to 8.5 with the maximum activity at pH 7.0, indicating that pH 7.0 is the optimum pH for this enzyme. In contrast, a recombinant glycerol dehydratase from the *K*. *pneumoniae* strain showed maximum activity at pH 8.5, and it lost more than 60% of its activity when the pH was below 7.5 [30, 31]. The pH stability of 2eGDHt was studied after it was preincubated at 4 °C for 24 h in various pH buffers (pH 4.2–9.0). As shown in Fig. 3D, 2eGDHt was stable over the pH range 6.0 to 8.0 with more than 40% relative activity after preincubation, whereas it was completely inactivated when the reaction pH was over 8.5. This constituted a great difference with that of a recombinant GDHt from *K*. *pneumoniae* 10,018, which retained less than 20% relative activity after preincubated at pH 6.0 for 240 min [23]. These results implied that 2eGDHt was more stable in a weakly acidic environment than the reported GDHt from *K*. *pneumoniae* strains. The above discrepancy in the biochemical characteristics of 2eGDHt compared with those of other *Klebsiella* glycerol dehydratases might be caused by the differences in their sequences and protein structures.

### Effects of metal ions and chemical reagents on the activity of 2eGDHt

To understand the effects of metal ions on the enzymatic activity of 2eGDHt, the enzyme activity was investigated with different metals (Ni^2+^, Cu^2+^, Co^2+^, Fe^3+^, Fe^2+^, Mn^2+^, Zn^2+^ Ca^2+^, and Na^+^) present at a concentration of 10 mM together with the glycerol substrate at 37 °C and pH 7.0. As shown in Fig. 3E, the enzymatic activity was inhibited by most of those metal ions to different extents, and the maximum inhibitory effect was achieved with Cu^2+^ which decreased the relative activity to 31.1%. However, the presence of Fe^2+^ increased the activity of 2eGDHt by 294%, but Fe^3+^ showed nearly no effect on 2eGDHt activity. These results implied that none of the detected metal ions were essential for the catalytic activity of 2eGDHt. Glycerol dehydratase is a metalloenzyme that requires K^+^ as the essential cofactor for catalysis. The introduction of Fe^2+^ might have caused changes in K^+^ coordination by 2eGDHt that maintained the proper position and orientation of the substrate during the reaction, while other metal ions might have had the opposite effect [32, 33].

The effects of various chemical reagents, including CTAB, Tween-20, EDTA, and SDS, on the activity of 2eGDHt were investigated at 37 °C and pH 7.0 with glycerol as the substrate. As shown in Fig. 3F, the presence of Tween-20 greatly increased the enzymatic activity of 2eGDHt by 290.2%. Similar effects of this chemical reagent on diol dehydratase have been reported [34, 35]. The stimulation of enzyme activity by Tween-20 might have resulted, because it increased the enzyme’s affinity for the substrate [36]. In contrast, EDTA and SDS decreased the enzyme activity to 51.2% and 36.2% relative activities, respectively. The presence of CTAB had nearly no effect on 2eGDHt activity. These results suggested that none of these chemical reagents was needed for the catalytic activity of 2eGDHt.

### Effects of the main impurities in crude glycerol on the activity of 2eGDHt

To study the tolerance of 2eGDHt against the crude glycerol environment, the effects of the main impurities in crude glycerol, including NaCl, methanol, oleic acid, and linoleic acid, on the activity of 2eGDHt were evaluated. As listed in Table 3, the presence of methanol at levels ranging from 7.5% (v/v) to 12.5% (v/v) significantly improved the activity of 2eGDHt by more than 10%, and it still retained more than 98% relative activity in the presence of 15% (v/v) methanol. The activity of 2eGDHt was enhanced by methanol at levels below 12.5% (v/v), which might be attributable to the interactions of the methanol with the hydrophobic amino acid residues around the catalytic site of 2eGDHt; this could have maintained the open conformation of the catalytic site during catalysis [37]. The introduction of NaCl at levels ranging from 2% (v/v) to 8% (v/v) showed little negative effect on the 2eGDHt activity which remained at more than 90% relative activity. The addition of oleic acid and linoleic acid showed obvious inhibitory effects on the activity of 2eGDHt, suggesting that these two impurities were the main factors affecting the enzyme activity. Nevertheless, more than 80% relative activity remained with these two impurities present at levels below 1% (v/v). In addition, the effects of mixtures of those impurities on the 2eGDHt activity were explored. The mixtures were prepared based on the percentage of each impurity found in crude glycerol (6% NaCl, 12% methanol, 0.5% oleic acid, and 0.5% linoleic acid) to mimic the crude glycerol environment. The activity of 2eGDHt was almost unaffected in the presence of the impurity mixture, and it retained more than 97% relative activity (Table 3). These results showed that 2eGDHt exhibited high tolerance against the crude glycerol impurities.

**Table 3:** Effects of various concentrations of main impurities of crude glycerol, including NaCl, methanol, oleic acid, and linoleic acid on the enzymatic activity of 2eGDHt

Impurity	Concentration (%, v/v)	Relative activity (%)
Control	0	100
NaCl	2	102.1 ± 2.8
	4	97.6 ± 2.5
	6	94.1 ± 3.2
	8	91.9 ± 3.4
Methanol	7.5	153.6 ± 2.2
	10	131.4 ± 1.5
	12.5	110.7 ± 2.9
	15	98.8 ± 3.1
Oleic acid	0.6	87.5 ± 3.4
	0.8	84.6 ± 2.8
	1.0	80.4 ± 1.7
	1.2	78.7 ± 2.6
Linoleic acid	0.6	83.4 ± 1.8
	0.8	80.8 ± 2.1
	1.0	78.2 ± 3.2
	1.2	70.3 ± 1.6
Mixture	6% NaCl, 12% methanol, 0.5% oleic acid and 0.5% linoleic acid	97.6 ± 2.3

The activity of 2eGDHt was determined by incubated with the addition of various concentrations of the impurities and the mixture for 10 min at 37 °C and pH 7.0. Enzyme activity without addition of impurity was taken as 100%. Results are represented mean ± SD from triplicate experiments

### Structural basis for the tolerance of 2eGDHt toward the crude glycerol impurities

**Fig. 4:**
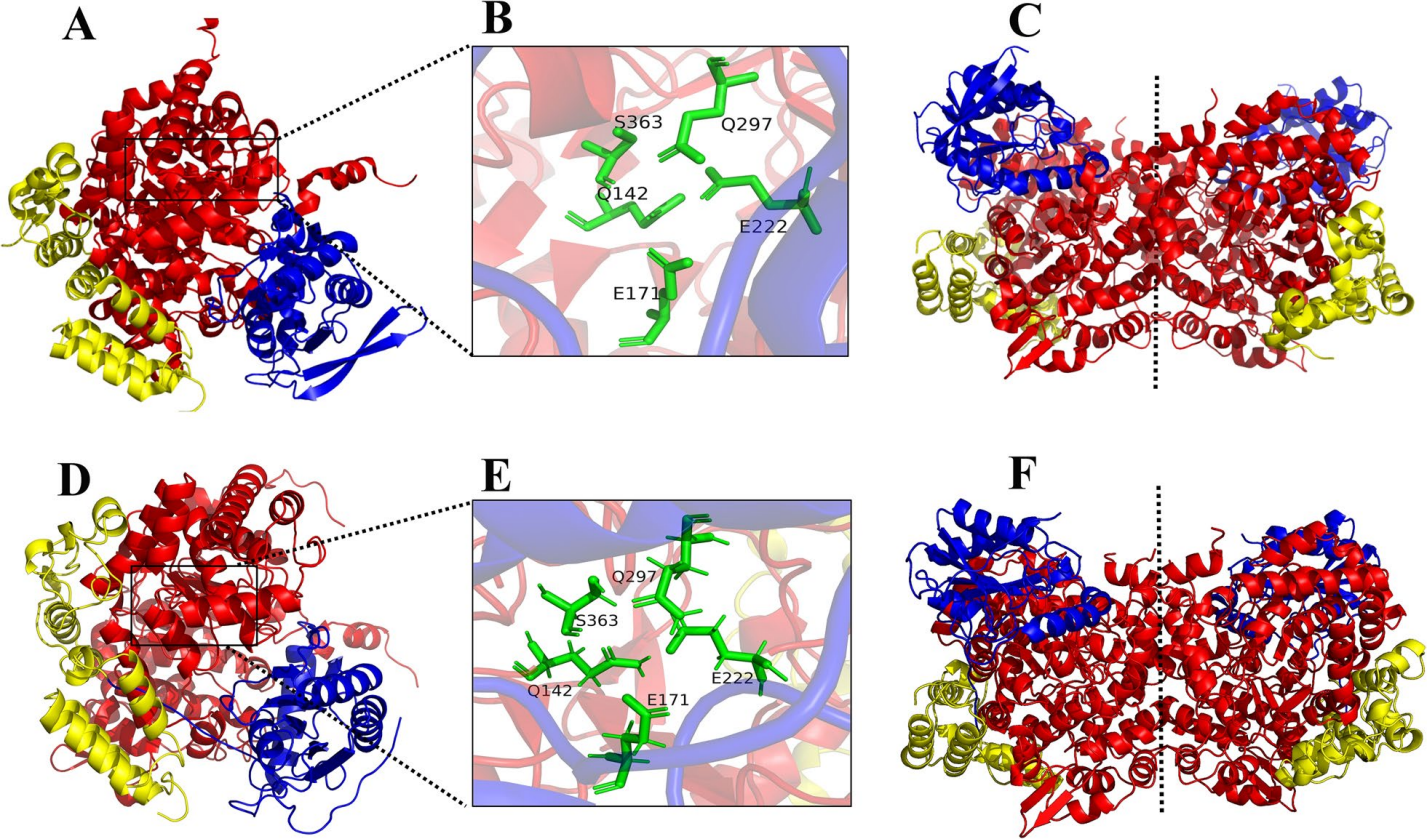
Comparison of the 3D structures for 2eGDHt and 1iwp-GDHt. The 3D structure of 2eGDHt was predicted with the AlphaFold server. **A**–**C** represent the *αβγ* heterotrimer, catalytic site, and dimer of 1iwp-GDHt respectively; **D**, **E** showed the predicted *αβγ* heterotrimer, catalytic site, and dimer of 2eGDHt respectively. The *α*, *β*, and *γ* subunits of GDHt are colored red, blue and yellow, respectively

To provide the protein structural basis of 2eGDHt, the three-dimensional (3D) structure of the protein was predicted with AlphaFold 2.1. As shown in Fig. 4, glycerol dehydratase (PDB ID: 1iwp) (1iwp-GDHt) from *K*. *pneumonia*, which showed the highest similarity (96.4%) with 2eGDHt, was used as the template for the model predictions. Compared with 1iwp-GDHt, the predicted 2eGDHt structure showed a very similar αβγ heterotrimer and its heterohexamer (*αβγ*)_2_ (see Additional file 1), which is a common feature of B_12_-dependent glycerol hydratases [27]. The two *αβγ* heterotrimers were connected only by the interactions of two α subunits in the (*αβγ*)_2_ heterohexamer. Meanwhile, the α subunit constitutes a (*β*/*α*)_8_ barrel, known as the triose-phosphate isomerase (TIM) barrel, located at the central part in both 1iwp-GDHt and 2eGDHt, which binds the substrate and the essential cofactor K^+^ [25]. Moreover, the active sites of 2eGDHt and 1iwp-GDHt were both located in the α subunit with five absolutely conserved catalytic amino acid residues, which was consistent with the multiple sequence alignment analysis results. These five catalytic amino acid residues formed a spatially independent local environment to protect highly active free radical intermediates during the catalysis process [38]. Considering the absolute conservation of the catalytic amino acid residues and high similarity of the predicted 3D structure models for 2eGDHt and other reported B_12_-dependent glycerol dehydratases from the *Klebsiella* genus, it is not surprising that the general biochemical characteristics, such as the substrate specificity, optimum catalytic temperature, and catalytic efficiency, of 2eGDHt were so similar to those of other reported B_12_-dependent glycerol dehydratases.

**Fig. 5:**
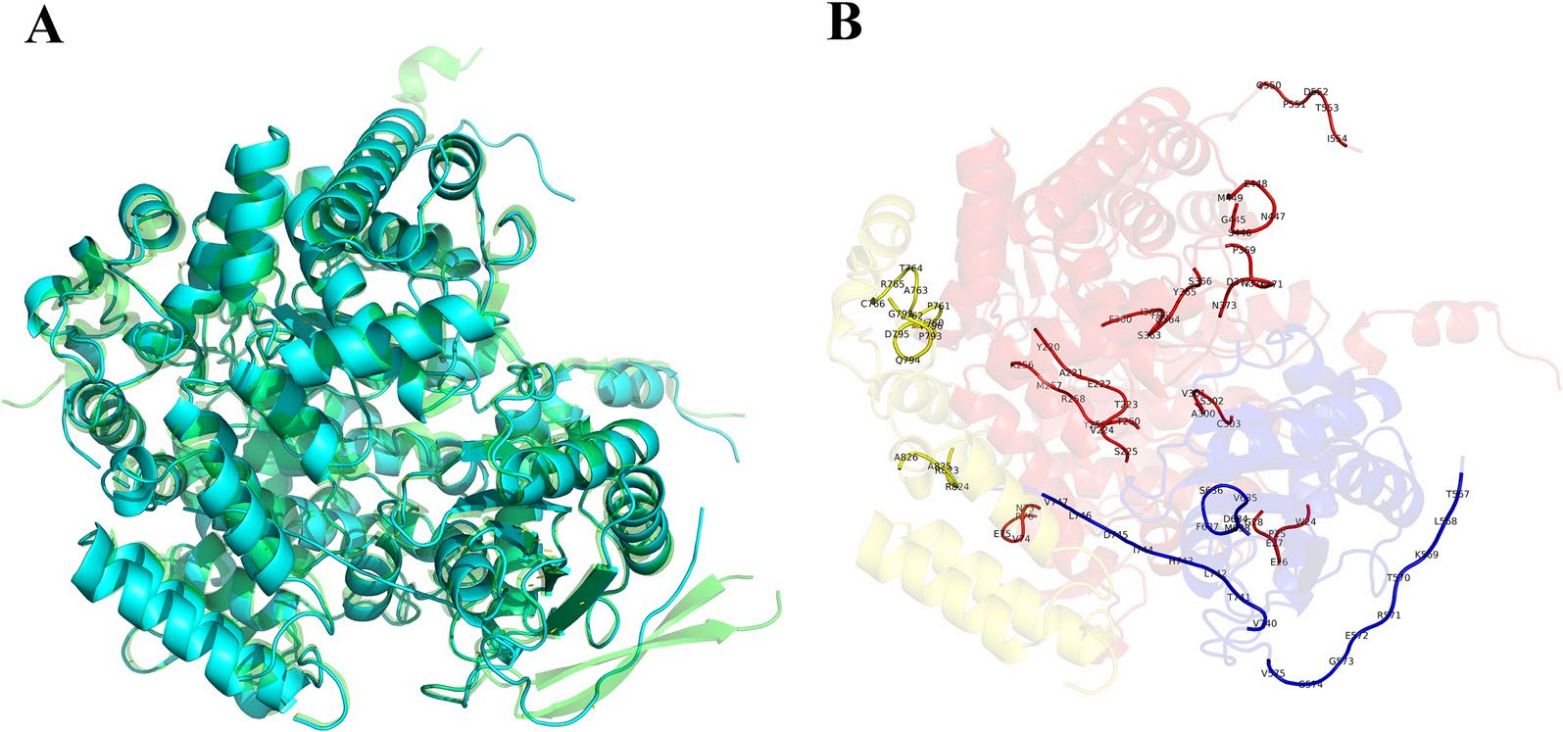
Comparative structural analysis of the coil regions in 2eGDHt and 1iwp-GDHt. **A** Superimoposition of the 2eGDHt (cyan) and 1iwp-GDHt (green). **B** The additional coil structures distributed in 2eGDHt

However, as shown in Fig. 5A, 2eGDHt contains more coil structures than 1iwp-GDHt at the monomer level. Someα-helix and β-strand structures in 1iwp-GDHt were replaced by coils in 2eGDHt. For example, two β-strands under the β subunit of 1iwp-GDHt were replaced by coil structures in 2eGDHt. Accordingly, as listed in Table 4, the proportion of coil structures and the ratio of coils to α-helixes in 2eGDHt were both the highest among those for the glycerol dehydratases compared. In addition, the additional coil structures were distributed in three subunits of 2eGDHt, while the α subunit contained most of those structures (Fig. 5B). Coils are the most flexible structures of proteins, and they often form irregular structures and cause large changes in the interactions with the solvent and ligands [39]. Most extreme enzymes contain more coil regions than general enzymes, which provides high flexibility in their overall structures and high catalytic activity in extreme environments [40]. The amplitude of the movements between secondary structures can be increased with more coils, resulting in higher structural flexibility [41]. This suggested that the abundance of coil regions in 2eGDHt might distinguish it from other glycerol dehydratases, which might contribute to the tolerance of 2eGDHt toward the crude glycerol environment. Coil structures are often involved in high variability across homologs, which leads to distinct characteristics among homologous proteins [42, 43]. Furthermore, 2eGDHt comes from the crude glycerol-tolerant strain *K*. *pneumoniae* 2e, which was isolated from a biodiesel-derived waste-contaminated soil environment and showed excellent performance in converting crude glycerol into 1,3-PDO. This may explain why the temperature and pH stabilities and the crude glycerol impurity tolerance of 2eGDHt were different from those of other glycerol dehydratases, albeit with highly similar sequences.

**Table 4:** Comparison of secondary structures proportions between 2eGHDt and other characterized glycerol dehydratases

Protein	*α*-Helix (%)	*β*-Sheet (%)	Coil (%)	Coil/*α*-helix
2eGDHt	41.1	13.8	45.1	1.10
1iwp-GDHt	42.2	13.0	44.8	1.06
1mmf-GDHt	41.6	13.6	44.7	1.07

The structures proportions were calculated based on the predicted 3D structure of 2eGHDt and crystal structure of 1iwp-GDHt (PDB ID: 1iwp) and 1mmf-GDHt (PDB ID: 1mmf) obtained from the RCSB PDB database

### Residue Iso744 in the extra coil region contributed to the tolerance of 2eGDHt against crude glycerol impurities by increasing the protein flexibility

**Fig. 6:**
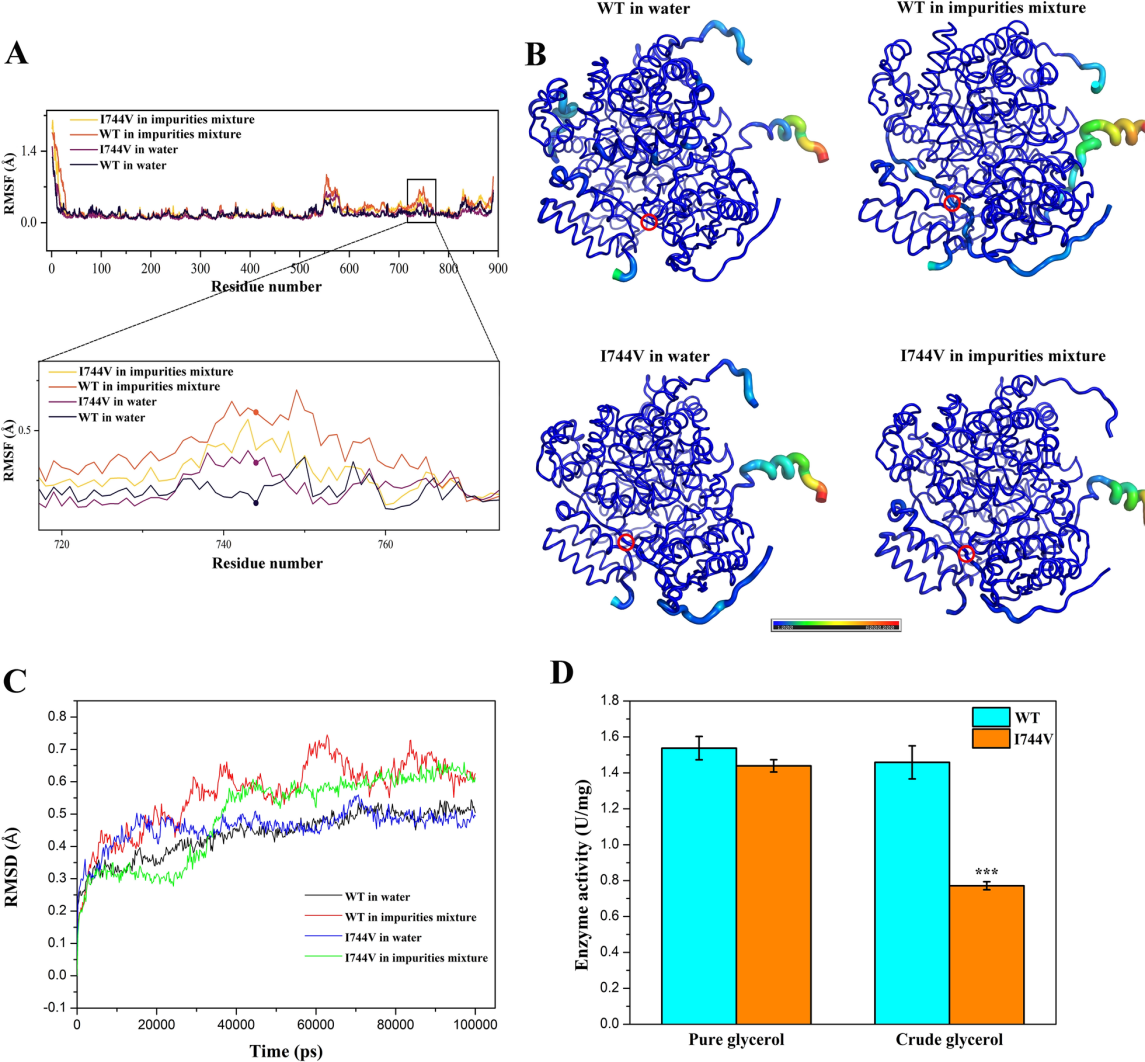
Comparison of 2eGDHt and mutant I744V. **A** Root-mean-square fluctuation (RMSF) analysis of 2eGDHt and mutant I744V under different conditions with molecular dynamics (MD) simulations. **B** Structures of 2eGDHt and mutant I744V colored by normalized RMSF values under different conditions. The thicker coils showed higher flexibility relative to other parts of the protein. The red circle indicates the residue isoleucine at position 744. **C** Root-mean-square deviation (RMSD) analysis of 2eGDHt and mutant I744V with molecular dynamics (MD) simulations under different conditions. **D** Effects of mutant I744V on the enzymatic activity of 2eGDHt in pure glycerol and crude glycerol. Asterisks indicate statistically significant differences compared to the WT (^***^p < 0.001, *n* = 3)

To study the effects of the key coil regions on the activity of 2eGDHt in the presence of crude glycerol impurities, a comparison of the root-mean-square fluctuations (RMSFs) of 2eGDHt in water and a mixture of impurities from crude glycerol was performed with molecular dynamic (MD) simulations. As shown in Fig. 6A and B, the RMSF values of 2eGDHt in the water and impurities mixture were generally comparable. The RMSF value of the region from residues 740 to 755, which contains one of the increased coil structures, was obviously higher for the mixture of impurities than for water. The residue at position 744 is isoleucine in 2eGDHt instead of valine, as in other glycerol dehydratases from *K*. *pneumonia* strains. This suggested that the unique residue Iso744 from one of the increased coil regions might play a critical role in the flexibility of 2eGDHt in the crude glycerol environment.

To investigate the role of residue Iso744 in the tolerance of 2eGDHt against crude glycerol impurities, site-directed mutagenesis of Iso744 was conducted. As shown in Fig. 5A and B, the RMSF value for the coil region from 740 to 755 in the mutant I744V was lower in the impurity mixture relative to wild-type (WT) 2eGDHt. This suggested that site-directed mutagenesis of I744V weakened the flexibility of 2eGDHt in the crude glycerol environment. In addition, as shown in Fig. 6C, the RMSD values for the mutant I744V and WT 2eGDHt showed no significant differences toward either the water or impurity mixture, indicating that the mutant of I744V has little impact on the stability of the overall structure of 2eGDHt.

Furthermore, the enzymatic activities of the I744V mutant were determined in pure and crude glycerol. As shown in Fig. 6D, the enzymatic activity of the I744V mutant showed no obvious differences from that of WT 2eGDHt in pure glycerol. The I744V mutation led to a great decrease in 2eGDHt activity, with a 52.8% relative activity of WT 2eGDHt in the crude glycerol environment, suggesting that the residue Iso744 plays a key role in the maintenance of protein flexibility and enzymatic activity and the tolerance of 2eGDHt against crude glycerol impurities. In addition, a structural analysis revealed that the residue Iso744 was far from the catalytic active site in 2eGDHt, and the I744V mutation did not affect the 2eGDHt activity in pure glycerol, indicating that the residue Iso744 has little impact on the stability of 2eGDHt.

## Conclusions

In this study, the novel glycerol dehydratase 2eGDHt from *K*. *pneumoniae* 2e with high tolerance against the main impurities in crude glycerol was characterized. Among various alcohol substrates, 2eGDHt showed the highest activity with glycerol, and *K*_*m*_ and *V*_*m*_ values were 3.42 mM and 58.15 nkat mg^−1^, respectively. It exhibited optimum activity at pH 7.0 and 37 °C. The enzyme was substantially activated by Fe^2+^ and Tween-20 with 294% and 290% relative activities, respectively. It was highly stable in the presence of various concentrations of the crude glycerol impurities and their mixtures, which mimicked the crude glycerol environment. The protein structure predicted for 2eGDHt contained more coil structures than those of reported glycerol dehydratases. The presence of a unique Val744 in one of the increased coil regions of 2eGDHt played a key role in its tolerance toward crude glycerol impurities by increasing the protein flexibility. This work furnishes insight into the enzymatic properties and molecular mechanism for tolerance of the novel glycerol dehydratase toward crude glycerol impurities, which is of great significance for the use of industrial glycerol dehydratase for 1,3-PDO production from crude glycerol.

## Methods

### Reagents, strains, plasmids, and culture conditions

Isopropyl-β-d-1-thiogalactopyranoside (IPTG) and 3-methyl-2-benzothiazolinone hydrazine (MBTH) were purchased from Macklin (Shanghai, China). A plasmid extraction kit and gel extraction kit were purchased from Tiangen Biotech and DongShengbio (Guangzhou, China), respectively. All restriction enzymes and DNA polymerases were purchased from Takara Biotechnology (Dalian, China). Polymerase chain reaction (PCR) primers were synthesized by Qinkebio (Changsha, China). All other reagents used were of molecular biology grade.

*K*. *pneumoniae* 2e was isolated from a soil sample collected from a biodiesel-derived waste-contaminated region in our previous study [24]. *Escherichia coli* DH5α and BL21 (DE3) cells purchased from Transgene (Beijing, China) were used for cloning and recombinant protein overexpression, respectively. The plasmid vector pET-28b purchased from Vazyme (Nanjing, China) was used for recombinant protein expression. *K*. *pneumoniae* 2e was cultured in nutrient broth (NB) medium at 37 °C and 170 rpm. *E*. *coli* was incubated in Luria–Bertani (LB) broth medium supplemented with 50 μg/mL kanamycin at 37 °C and 200 rpm.

### Bioinformatic analysis

Sequence similarities were detected with the NCBI BLAST algorithm. Phylogenetic analyses was carried out with MEGA 5.1 using the neighbor-joining algorithm with bootstrap values. Multiple sequence alignment was conducted with Clustalx and GeneDoc software based on the protein sequence of glycerol dehydratase from the *Klebsiella* genus. Homology modeling of glycerol dehydratase from *K*. *pneumoniae* 2e was performed with a version of AlphaFold 2.1 [44]. The homodimer structure of glycerol dehydratase from *K*. *pneumoniae* 2e was predicted with Gramm-X and PDBePISA software [45, 46]. All protein structures were visualized with PyMOL software.

### Cloning of the gene-encoding glycerol dehydratase of *K*. *pneumoniae* 2e

The genomic DNA of *K*. *pneumoniae* 2e used as a template for amplification of the gene-encoding glycerol dehydratase was extracted as previously described [24]. The primers used for glycerol dehydratase gene *dhaB* amplification are listed in Additional file 1: Table S1. PCR was conducted with PrimeSTAR HS DNA polymerase (TAKARA, Kyoto, Japan) according to the manufacturer’s instructions. The amplified PCR products were purified with a gel extraction kit (Tiangen, Beijing, China) and subsequently inserted into the vector pET-28b after double restriction enzyme digestion and ligation with T4 DNA ligase. The recombinant plasmid was transformed into *E*. *coli* BL21 (DE3) cells for the overexpression of 2eGDHt.

### Site-directed mutagenesis

Site-directed mutation of 2eGDHt was conducted with the Site-directed Mutagenesis Kit (Sangon Biotech, Shanghai, China) according to the manufacturer’s instructions. The primers used for site-directed mutagenesis are listed in Additional file 1: Table S1. The recombinant plasmid containing the desired mutation was screened and identified by DNA sequencing, and it was subsequently transformed into *E*. *coli* BL21 (DE3) cells for recombinant protein overexpression.

### Overexpression and purification of recombinant 2eGDHt

The recombinant *E*. *coli* BL21 (DE3) were inoculated into 100 mL of LB liquid medium with 50 μg/mL kanamycin at 37 °C until the OD_600_ of the cell reached 0.6. The protein was induced by the addition of 0.8 mM IPTG and was further incubated for 4 h at 37 °C. Cells were collected by centrifugation at 4 °C and 12,000*g* for 10 min and washed twice with PBS buffer (0.05 mM, pH 7.2). The cells were resuspended in lysis buffer (50 mM Tris–HCl, 100 mM NaCl) and disrupted by sonication for 5 min at 4 °C. The supernatant was harvested after centrifugation at 12,000*g* and 4 °C for 15 min. The recombinant protein containing a His-tag in the collected supernatant was purified by Ni–NTA resin (Smart-Lifesciences, Changzhou, China) according to the manufacturer’s instructions. The purified proteins were analyzed by SDS-PAGE, and the protein concentration was measured with a BCA protein assay kit (Tiangen Bioetech, Beijing, China).

### Enzymatic assay

The glycerol dehydratase activity was determined with the 3-methyl-2-benzothiazolinone hydrazine (MBTH) method [47]. Briefly, the assay mixture (1 mL) contained 0.2 M 1,2-PDO, 0.05 M KCl, 35 mM PBS buffer (pH 8.0), 15 μM coenzyme B_12_, and an appropriate amount of the enzyme samples. After incubation at 37 °C for 10 min, the enzyme reaction was terminated by the addition of 500 μL of 0.1% MBTH and incubation at 37 °C for 15 min. Subsequently, 1 mL of double-distilled H_2_O was added, and the amount of aldehyde formed was determined from the absorbance at 305 nm. The enzyme activity needed to produce 1 μM aldehyde under the assay conditions was defined as one unit of enzyme activity.

### Biochemical characterization of 2eGDHt

The substrate specificity of the enzyme was investigated with 0.2 M glycerol, 1,2-propanediol, 1,2-butanediol, butanol, methanol, ethanol, and isopropanol used as the substrates. The enzyme activities against different substrates were determined at 37 °C and pH 7.0.

To assay the kinetic parameters of 2eGDHt for glycerol and 1,2-PDO, the enzyme was incubated with various substrate concentrations (2 to 200 mM) at the optimum temperature (37 °C) and pH (7.0). The Michaelis constant (*K*_m_), maximum velocity (*V*_max_), and turnover number (*K*_cat_) were obtained from the Michaelis–Menten equation in Lineweaver–Burk plots. The catalytic efficiencies were calculated with *K*_cat_/*V*_max_.

The effects of temperature and pH on the activity of 2eGDHt were determined by estimating the enzyme activities at various temperatures and pH values ranging from 4–68 °C and pH 4.2–9.0, respectively.

The temperature and pH stability of 2eGDHt was detected by preincubating the enzyme without substrate at various temperatures and pH, ranging from 4 to 68 °C and pH 4.2–9.0, respectively, and estimating the residual activity under standard assay conditions.

The effects of metal ions on the enzymatic activity of 2eGDHt were tested by adding various metal ions (Mg^2+^, Ca^2+^, Cu^2+^, Mn^2+^, Zn^2+^, Fe^2+^, Fe^3+^, and Ba^2+^) and chemical reagents (CTAB, Tween-20, EDTA, and SDS) to give final concentrations of 10 mM in the reaction mixture.

### Effects of the main impurities in crude glycerol on 2eGDHt activity

The effect of the main impurities in crude glycerol, including methanol, NaCl, oleic acid, and linoleic acid, on the enzymatic activity of 2eGDHt were investigated. The individual effects for various concentrations of the impurities on enzyme activity were determined as follows (wt/wt): NaCl was added at 2%, 4%, 6%, and 8%; methanol was added at 7.5%, 10%, 12.5%, and 15%; and oleic acid and linoleic acid were added at 0.6%, 0.8%, 1.0%, and 1.2% [24]. The mixture of those impurities was prepared based on the percentage of each impurity in crude glycerol, i.e., 6% NaCl, 12% methanol, 0.5% oleic acid, and 0.5% linoleic acid [24]. Glycerol was used as the substrate in the above enzyme assays. The activity obtained without the additives was used as the control and taken as 100%.

### Molecular dynamics (MD) simulations

The MD simulations were conducted with the GROMACS 2022.2 software and the GROMOS CHARMM36m force field [48]. The protein structure was solvated with the TIP3P water molecule model in cubic boxes with a solute-wall minimum distance of 10 Å [49]. The antechamber, parmchk2, and acpype programs were used to generate protein and impurity mixture topology files. Then, the protein and impurity mixture topology files were concatenated to create a protein–impurities mixture topology file. The impurity mixture, including 6% NaCl, 12% methanol, 0.5% oleic acid, and 0.5% linoleic acid, was inserted into a topology file to construct a mimic system for the crude glycerol impurity environment. Potassium and sodium ions were added to the virtual water box as counter ions to neutralize the overall system charge. Electrostatic interactions are calculated using the Particle Mesh Ewald algorithm [50]. The system was submitted to energy minimization using the steepest descent algorithm for 10,000 steps prior to MD simulation. Subsequently, the 125 ps NVT ensemble was conducted at temperatures close to 298 K. Then, a location with positional constraints on the protein was selected for a 125 ps NPT ensemble for balancing, followed by a production simulation run (330 K, 1 bar, dt = 0.002, steps = 1,500,000). During the MD operation, the coordinates, energy, and velocity were stored every 1 ns and used for trajectory analyses. The dynamic changes in the RMSF values were analyzed by the GROMACS tool.

### Supplementary Information

**Additional file 1: Table S1.** Primers used for PCR in this study.

## Data Availability

All data generated or analyzed during this study are included in this published article and its supplementary information file.

## References

[1] Zhang J, Wang Y, Muldoon VL, Deng S (2022). Crude glycerol and glycerol as fuels and fuel additives in combustion applications. Renew Sustain Energy Rev.

[2] Kumar LR, Yellapu SK, Tyagi RD, Zhang X (2019). A review on variation in crude glycerol composition, bio-valorization of crude and purified glycerol as carbon source for lipid production. Bioresour Technol.

[3] Samul D, Leja K, Grajek W (2014). Impurities of crude glycerol and their effect on metabolite production. Ann Microbiol.

[4] Sivasankaran C, Ramanujam PK, Balasubramanian B, Mani J (2016). Recent progress on transforming crude glycerol into high value chemicals: a critical review. Biofuels.

[5] Clomburg JM, Gonzalez R (2013). Anaerobic fermentation of glycerol: a platform for renewable fuels and chemicals. Trends Biotechnol.

[6] Chen Z, Liu D (2016). Toward glycerol biorefinery: metabolic engineering for the production of biofuels and chemicals from glycerol. Biotechnol Biofuels.

[7] Dobson R, Gray V, Rumbold K (2012). Microbial utilization of crude glycerol for the production of value-added products. J Ind Microbiol Biotechnol.

[8] Crosse AJ, Brady D, Zhou N, Rumbold K (2019). Biodiesel's trash is a biorefineries' treasure: the use of "dirty" glycerol as an industrial fermentation substrate. World J Microbiol Biotechnol.

[9] Mu Y, Teng H, Zhang DJ, Wang W, Xiu ZL (2006). Microbial production of 1,3-propanediol by *Klebsiella pneumoniae* using crude glycerol from biodiesel preparations. Biotechnol Lett.

[10] Celinska E (2012). *Klebsiella* spp as a 1,3-propanediol producer: the metabolic engineering approach. Crit Rev Biotechnol.

[11] Venkataramanan KP, Boatman JJ, Kurniawan Y, Taconi KA, Bothun GD, Scholz C (2012). Impact of impurities in biodiesel-derived crude glycerol on the fermentation by *Clostridium pasteurianum* ATCC 6013. Appl Microbiol Biotechnol.

[12] Chatzifragkou A, Papanikolaou S (2012). Effect of impurities in biodiesel-derived waste glycerol on the performance and feasibility of biotechnological processes. Appl Microbiol Biotechnol.

[13] Jiang W, Wang S, Wang Y, Fang B (2016). Key enzymes catalyzing glycerol to 1,3-propanediol. Biotechnol Biofuels.

[14] Daniel R, Bobik TA, Gottschalk G (1998). Biochemistry of coenzyme B_12_-dependent glycerol and diol dehydratases and organization of the encoding genes. FEMS Microbiol Rev.

[15] Nasir A, Ashok S, Shim JY, Park S, Yoo TH (2020). Recent progress in the understanding and engineering of coenzyme B_12_-dependent glycerol dehydratase. Front Bioeng Biotechnol.

[16] Liu JZ, Xu W, Chistoserdov A, Bajpai RK (2016). Glycerol dehydratases: biochemical structures, catalytic mechanisms, and industrial applications in 1,3-propanediol production by naturally occurring and genetically engineered bacterial strains. Appl Biochem Biotechnol.

[17] Kajiura H, Mori K, Tobimatsu T, Toraya T (2001). Characterization and mechanism of action of a reactivating factor for adenosylcobalamin-dependent glycerol dehydratase. J Biol Chem.

[18] Wong CL, Huang CC, Chen WM, Chang JS (2011). Converting crude glycerol to 1,3-propandiol using resting and immobilized *Klebsiella* sp. HE-2 cells. Biochem Eng J.

[19] Kong DS, Park EJ, Mutyala S, Kim M, Cho Y, Oh SE, Kim C, Kim JR (2021). Bioconversion of crude glycerol into 1,3-propanediol (1,3-PDO) with bioelectrochemical system and zero-valent iron using *Klebsiella pneumoniae* L17. Energies.

[20] Yang X, Kim DS, Choi HS, Kim CK, Thapa LP, Park C, Kim SW (2017). Repeated batch production of 1,3-propanediol from biodiesel derived waste glycerol by *Klebsiella pneumoniae*. Chem Eng J.

[21] Laura M, Monica T, Dan-Cristian V (2020). The effect of crude glycerol impurities on 1,3-propanediol biosynthesis by *Klebsiella pneumoniae* DSMZ 2026. Renew Energy.

[22] Xu X, Zhang G, Wang L, Ma B, Li C (2009). Quantitative analysis on inactivation and reactivation of recombinant glycerol dehydratase from *Klebsiella pneumoniae* XJPD-Li. J Mol Catal B Enzym.

[23] Qi X, Guo Q, Wei Y, Xu H, Huang R (2012). Enhancement of pH stability and activity of glycerol dehydratase from *Klebsiella pneumoniae* by rational design. Biotechnol Lett.

[24] Ma J, Jiang H, Hector SB, Xiao Z, Li J, Liu R, Li C, Zeng B, Liu GQ, Zhu Y (2019). Adaptability of *Klebsiella pneumoniae* 2e, a newly isolated 1,3-propanediol-producing strain, to crude glycerol as revealed by genomic profiling. Appl Environ Microbiol.

[25] Yamanishi M, Yunoki M, Tobimatsu T, Sato H, Matsui J, Dokiya A, Iuchi Y, Oe K, Suto K, Shibata N (2002). The crystal structure of coenzyme B_12_-dependent glycerol dehydratase in complex with cobalamin and propane-1,2-diol. Eur J Biochem.

[26] Macis L, Daniel R, Gottschalk G (1998). Properties and sequence of the coenzyme B_12_-dependent glycerol dehydratase of *Clostridium pasteurianum*. FEMS Microbiol Lett.

[27] Liao DI, Dotson G, Turner IJ, Reiss L, Emptage M (2003). Crystal structure of substrate free form of glycerol dehydratase. J Inorg Biochem.

[28] Toraya T, Tanokuchi A, Yamasaki A, Nakamura T, Ogura K, Tobimatsu T (2016). Diol dehydratase-reactivase is essential for recycling of coenzyme B_12_ in diol dehydratase. Biochemistry.

[29] Sankaranarayanan M, Seol E, Kim Y, Chauhan AS, Park S (2017). Measurement of crude-cell-extract glycerol dehydratase activity in recombinant *Escherichia coli* using coupled-enzyme reactions. J Ind Microbiol Biotechnol.

[30] Wang F, Qu H, Tian P, Tan T (2007). Heterologous expression and characterization of recombinant glycerol dehydratase from *Klebsiella pneumoniae* in *Escherichia coli*. Biotechnol J.

[31] Xu XL, Zhang GL, Lv B, Yuan YJ, Li C (2011). Recombinant glycerol dehydratase from *Klebsiella pneumonia* XJPD-Li: induction optimization, purification and characterization. Appl Biochem Biotechnol.

[32] Gohara DW, Di Cera E (2016). Molecular mechanisms of enzyme activation by monovalent cations. J Biol Chem.

[33] Ragsdale SW (2006). Metals and their scaffolds to promote difficult enzymatic reactions. Chem Rev.

[34] Wei X, Meng X, Chen Y, Wei Y, Du L, Huang R (2014). Cloning, expression, and characterization of coenzyme-B_12_-dependent diol dehydratase from *Lactobacillus diolivorans*. Biotechnol Lett.

[35] Toraya T, Honda S, Mori K (2010). Coenzyme B_12_-dependent diol dehydratase is a potassium ion-requiring calcium metalloenzyme: evidence that the substrate-coordinated metal ion is calcium. Biochemistry.

[36] Gupta P, Mishra AK, Vakhlu J (2017). Cloning and characterization of thermo-alkalistable and surfactant stable endoglucanase from Puga hot spring metagenome of Ladakh (J&K). Int J Biol Macromol.

[37] Patel V, Nambiar S, Madamwar D (2014). An extracellular solvent stable alkaline lipase from *Pseudomonas* sp. DMVR46: partial purification, characterization and application in non-aqueous environment. Process Biochem.

[38] Bilic L, Baric D, Banhatti RD, Smith DM, Kovacevic B (2019). Computational study of glycerol binding within the active site of coenzyme B_12_-dependent diol dehydratase. J Phys Chem B.

[39] Bhatt HB, Singh SP (2020). Cloning, expression, and structural elucidation of a biotechnologically potential alkaline serine protease from a newly isolated Haloalkaliphilic *Bacillus lehensis* JO-26. Front Microbiol.

[40] Nestl BM, Hauer B (2014). Engineering of flexible loops in enzymes. ACS Catal.

[41] Malabanan MM, Amyes TL, Richard JP (2010). A role for flexible loops in enzyme catalysis. Curr Opin Struct Biol.

[42] Furnham N, Sillitoe I, Holliday GL, Cuff AL, Laskowski RA, Orengo CA, Thornton JM (2012). Exploring the evolution of novel enzyme functions within structurally defined protein superfamilies. PLoS Comput Biol.

[43] Yu H, Yan Y, Zhang C, Dalby PA (2017). Two strategies to engineer flexible loops for improved enzyme thermostability. Sci Rep.

[44] Jumper J, Evans R, Pritzel A, Green T, Figurnov M, Ronneberger O, Tunyasuvunakool K, Bates R, Zidek A, Potapenko A (2021). Highly accurate protein structure prediction with AlphaFold. Nature.

[45] Tovchigrechko A, Vakser IA (2006). GRAMM-X public web server for protein-protein docking. Nucleic Acids Res.

[46] Krissinel E, Henrick K (2007). Inference of macromolecular assemblies from crystalline state. J Mol Biol.

[47] Zhang G, Ma B, Xu X, Li C, Wang L (2007). Fast conversion of glycerol to 1,3-propanediol by a new strain of *Klebsiella pneumoniae*. Biochem Eng J.

[48] Abraham MJ, Murtola T, Schulz R, Páll S, Smith JC, Hess B, Lindahl E (2015). GROMACS: High performance molecular simulations through multi-level parallelism from laptops to supercomputers. SoftwareX.

[49] Mark P, Nilsson L (2001). Structure and dynamics of the TIP3P, SPC, and SPC/E water models at 298 K. J Phys Chem A.

[50] Qian X, Schlick T (2002). Efficient multiple-time-step integrators with distance-based force splitting for particle-mesh-Ewald molecular dynamics simulations. J Chem Phys.

