# Supplementary material for: Biochemical and molecular characterization of a novel glycerol dehydratase from Klebsiella pneumoniae 2e with high tolerance against crude glycerol impurities
Source: Biotechnol Biofuels Bioprod. 2023 Nov 16;16:175. doi: 10.1186/s13068-023-02427-8 (PMC10655381; doi:10.1186/s13068-023-02427-8)
Supplement: Supplementary file 1 — Additional file 1: Table S1. Primers used for PCR in this study. [file 13068_2023_2427_MOESM1_ESM.docx]

**Table S1** Primers used for PCR in this study

| Item | Sequence | |
| --- | --- | --- |
|  | **5’** | **3’** |
| *dhaB* | CCGGAATTCGATGAAAAGATCAAAACGATTTGCAG | CCCAAGCTTTTAGCTTCCTTTACGCAGCTTATGC |
| *I744V* | CCCTGCACgtcGACTTAGTAAGGGAGTGACCAT | TAAGTCgacGTGCAGGGTGACGGGCTCG |

Underlined indicated the position of mutant amino acid.
